# The impact of continuous cultivation of *Ganoderma lucidum* on soil nutrients, enzyme activity, and fruiting body metabolites

**DOI:** 10.1038/s41598-024-60750-y

**Published:** 2024-05-02

**Authors:** Wei Ji, Ni Zhang, Wenying Su, Xia Wang, Xiaomei Liu, Yipu Wang, Kelong Chen, Likai Ren

**Affiliations:** 1 Qinghai Province Key Laboratory of Physical Geography and Environmental Process, College of Geographical Science, Qinghai Normal University, Xining, 810008 China; 2 Lianyungang Academy of Agricultural Sciences, Lianyungang, 222006 China; 3 Key Laboratory of Tibetan Plateau Land Surface Processes and Ecological Conservation (Ministry of Education), Qinghai Normal University, Xining, 810008 China; 4 National Positioning Observation and Research Station of Qinghai Lake Wetland Ecosystem in Qinghai, National Forestry and Grassland Administration, Haibei, 812300 China

**Keywords:** Microbiology, Fungi

## Abstract

To explore the impacts of continuous *Ganoderma lucidum* cultivation on soil physicochemical factors, soil enzyme activity, and the metabolome of *Ganoderma lucidum* fruiting bodies, this study conducted two consecutive years of cultivation on the same plot of land. Soil physicochemical factors and enzyme activity were assessed, alongside non-targeted metabolomic analysis of the *Ganoderma lucidum* fruiting bodies under continuous cultivation. The findings unveiled that in the surface soil layer (0–15 cm), there was a declining trend in organic matter, ammonium nitrogen, available phosphorus, available potassium, pH, polyphenol oxidase, peroxidase, alkaline phosphatase, and sucrase, whereas nitrate nitrogen, electrical conductivity (EC), and salt content exhibited an upward trend. Conversely, in the deeper soil layer (15–30 cm), organic matter, ammonium nitrogen, available potassium, alkaline phosphatase, and sucrase demonstrated a decreasing trend, while nitrate nitrogen, available phosphorus, pH, EC, salt content, polyphenol oxidase, and soil peroxidase showed an increasing trend. Metabolomic analysis of *Ganoderma lucidum* fruiting bodies distinguished 64 significantly different metabolites between the GCK and GT groups, with 39 components having markedly higher relative contents in GCK and 25 components having significantly lower relative contents in GCK compared to GT. Moreover, among these metabolites, there were more types with higher contents in the fruiting bodies harvested in the first year (GCK) compared to those harvested in the second year (GT), with pronounced differences. KEGG pathway analysis revealed that GCK exhibited more complex metabolic pathways compared to GT. The metabolites of *Ganoderma lucidum* fruiting bodies were predominantly influenced by soil physicochemical factors and soil enzyme activity. In the surface soil layer (0–15 cm), the metabolome was significantly affected by soil pH, soil organic matter, available phosphorus, and soil alkaline phosphatase, while in the deeper soil layer (15–30 cm), differences in the *Ganoderma lucidum* metabolome were more influenced by soil alkaline phosphatase, soil catalase, pH, nitrate nitrogen, and soil sucrase.

## Introduction

*Ganoderma lucidum*, commonly known as Lingzhi in Chinese or Reishi in Japanese, is a traditional medicinal mushroom that has been utilized for centuries in Asian countries like China, Japan, and Korea, as well as in various other regions, for the prevention and treatment of diverse ailments^1,2^. *Ganoderma lucidum* is abundant in pharmacologically active compounds and is devoid of toxic side effects. Its bioactive constituents encompass Ganoderma polysaccharides, peptides, triterpenoids, and 18 amino acids^3^. *Ganoderma lucidum* has been documented to mitigate central nervous system excitability and exhibit specific analgesic properties^4^. Moreover, it showcases detoxifying, hypoglycemic, immunomodulatory, anti-tumor, and radioprotective attributes^5,6^. Additionally, *Ganoderma lucidum* holds substantial cultural and ornamental significance, being traditionally revered as a symbol of fortune and affluence, and can be cultivated into visually appealing artifacts^7^.

In recent years, continuous cultivation of *Ganoderma lucidum* in the same area has led to slow growth, smaller and more malformed fruiting bodies, and decreased yields, causing significant economic losses to *Ganoderma lucidum* growers^8^. Many growers opt to change *Ganoderma lucidum* cultivation sites every two years, which increases cultivation costs. Soil nutrition is a crucial factor in *Ganoderma lucidum* continuous cropping obstacles, as high nutrient levels in the soil may lead to the accumulation of antagonistic fungi such as *Trichoderma* and *Mucor*, thus exacerbating cultivation issues^1^. Non-targeted metabolomics approaches have been widely employed in various aspects of *Ganoderma lucidum* research, including metabolite identification and functional studies^9^, quality control and product evaluation^10^, screening and development of bioactive compounds^11^, and elucidation of biosynthetic pathways^12^. However, the impact of continuous cultivation on the fruiting body metabolome of *Ganoderma lucidum* and the relationships between soil physicochemical factors, enzyme activities, and *Ganoderma lucidum* fruiting body metabolomes remain unclear. Therefore, this study aims to investigate the effects of continuous *Ganoderma lucidum* cultivation on soil physicochemical factors, enzyme activities, and *Ganoderma lucidum* fruiting body metabolomes by conducting two years of consecutive cultivation in the same land. The study will analyze the correlations between them to provide data support for further soil improvement to mitigate *Ganoderma lucidum* continuous cropping obstacles.

## Materials and methods

### *Ganoderma lucidum* cultivation

The Ganoderma strain used in this study is Yunzhi No.1 bred by the Agricultural Science Institute of Lianyungang City. The cultivation method for Ganoderma adopts the log cultivation method. The mother culture of Ganoderma in slant tubes is cultured on Potato Dextrose Agar (PDA) medium (potatoes 200 g, glucose 20 g, agar 15 g, water 1 L). 3–4 pieces of 5 mm*5 mm fungal pieces are inoculated into liquid starter medium using an inoculation needle. The liquid starter medium is Potato Glucose Liquid medium (potatoes 200 g, glucose 20 g, water 1L). The cultivation conditions are 25 °C and 160 rpm for 3–4 days. The fermented Ganoderma liquid starter is inoculated into sterilized solid substrate medium at a rate of 3–5% (formula: sawdust 78%, bran 13%, corn flour 7%, sucrose 1%, gypsum 1%, and appropriate water to adjust the pH, pH is naturally adjusted). Cultivation is carried out at 25 °C in the dark for 25–35 days until the mycelium fully colonizes the cultivation substrate. For log cultivation, fresh oak branches from Lianyungang area with diameters of 5–10 cm are selected and cut into 30 cm long segments, bundled in groups of three, and placed into cultivation bags. Additionally, 200 g of sawdust with a moisture content of 50% is added to each bag. The bags are then subjected to high-pressure steam sterilization at 121 °C for 2 h. Inoculate the prepared solid substrate mentioned above at a rate of 3–5% with the sterilized log cultivation medium, and then cultivated in the dark for 25–30 days until the mycelium colonizes the entire bag. Once the Ganoderma mycelium has fully colonized the log segments, they are transferred to the Ganoderma cultivation mushroom house and buried in soil, leaving 2 cm exposed above the ground. Subsequently, cultivation management is carried out following the standardized procedures described by Diego^13^.

### Soil sample and fruiting body sample collection

The selected *Ganoderma lucidum* cultivation chambers were initially established in May 2021 for the first cultivation cycle, followed by consecutive cultivation in May 2022 for the second year. Soil samples were collected in May 2021 before the cultivation of *Ganoderma lucidum*. Soil samples were collected using a soil auger with a diameter of 4.5 cm, employing a five-point sampling method in each plot. Samples were taken from the 0–30 cm soil layer (0–15 cm and 15–30 cm). The soil from the same layer was mixed thoroughly and sieved for determination of soil physicochemical factors and enzyme activity. Three replicates were set for each sample. The soil samples collected were labeled as GCK11 (0–15 cm topsoil sample), GCK12 (15–30 cm subsoil sample). In May 2022, before the cultivation of *Ganoderma lucidum*, soil samples were collected using the same method as described above. Soil samples were labeled as GT11 (0–15 cm topsoil sample) and GT12 (15–30 cm subsoil sample). Three replicates were also set for each sample.

The *Ganoderma lucidum* fruiting body samples selected for analysis were harvested in October 2021 from the first year of cultivation (sample named GCK) and in October 2022 from the consecutive second year of cultivation (sample named GT). Metabolomic analysis was conducted on the *Ganoderma lucidum* fruiting bodies, with three replicates set for each sample.

### Experimental design

#### Determination of soil physicochemical factors and enzyme activity

The soil samples were naturally dried in a light-shielded room, followed by the removal of sand, stones, and plant residues. The dried soil was then sieved through a 40-mesh sieve. Subsequently, the sieved soil samples were subjected to the determination of organic matter content, ammonium nitrogen content, nitrate nitrogen content, available phosphorus content, rapidly available potassium content, electrical conductivity (EC), salt content and pH^14–16^.

The content of polyphenol oxidase, catalase, sucrase, and alkaline phosphatase was determined using assay kits from Suzhou Grace Biotechnology Co., Ltd. The kit numbers are G0311F, G0303F48, G0302F48, and G0305F for polyphenol oxidase, catalase, sucrase, and alkaline phosphatase, respectively.

#### Metabolomic analysis of *Ganoderma lucidum* fruiting bodies

##### Sample extraction

After slow thawing of the samples at 4 °C, weigh an appropriate amount of sample (50–100 mg) and add 1 ml of water: acetonitrile: isopropanol (1:1:1, v/v). Vortex for 60 s, then sonicate at low temperature for 30 min. Centrifuge at 12,000 rpm for 10 min at 4 °C and collect the supernatant. Place the supernatant at − 20 °C for 1 h to precipitate proteins. Centrifuge again at 12,000 rpm for 10 min at 4 °C, then collect the supernatant and dry it under vacuum. Reconstitute in 200 μL 30% ACN, vortex, and centrifuge at 14,000 rpm for 15 min at 4 °C. Finally, collect the supernatant for further analysis.

##### Liquid chromatography parameters

Instrument: Vanquish UPLC (Thermo, USA), column: Waters HSS T3 (100*2.1 mm, 1.8 μm), mobile phase: solvent A: 0.1% formic acid in water, solvent B: 0.1% formic acid in acetonitrile, flow rate: 0.3 ml/min, column temperature: 40 °C, injection volume: 2 μl, gradient elution: 0.0–1.0 min, B maintained at 0% , 1.0–9.0 min, B linearly increased from 0 to 95%, 9.0–13.0 min, B maintained at 95%, 13.0–13.1 min, B linearly decreased from 95 to 0%, 13.1–17.0 min, B maintained at 0%. Throughout the entire analysis, samples were maintained at 4 °C in the autosampler. To mitigate the impact of fluctuations in instrument detection signals, samples were analyzed in random order. QC samples were inserted into the sample queue to monitor and evaluate the stability of the system and the reliability of the experimental data.

### Mass spectrometry conditions

Instrument: Thermo Q Exactive HFX high-resolution mass spectrometer (Thermo Fisher Scientific, USA), ionization mode: electrospray ionization (ESI), sheath gas: 40arb, auxiliary gas: 10arb, spray voltage: 3000 V (positive ionization mode)/− 2800 V (negative ionization mode), temperature: 350 °C, Ion transfer tube temperature: 320 °C, scan mode: Full-MS-ddMS2 (Data-dependent MS/MS), scan type: positive ion mode/negative ion mode, MS1 Scan Range: 70–1050 Da, MS2 scan range: 200–2000 Da, MS1 Resolution: 70,000, MS2 Resolution: 17,500.

### Data analysis workflow

Origin 9.1 software was chose for analysis the acquired data. SPSS software was employed to analyze the significance of inter-group differences, with a significance threshold set at *P* < 0.05. Photoshop software was utilized to process images. Multivariate Analysis: Canoco 5.0 software was chose for multivariate analysis.

The raw metabolomics data were processed using the metabolomics software Progenesis QI (Waters Corporation, Milford, USA) for baseline filtering, peak identification, integration, retention time correction, and peak alignment. This process resulted in a data matrix containing retention time, mass-to-charge ratio, and peak intensity. The main databases used for metabolite identification include http://www.hmdb.ca/ and https://metlin.scripps.edu/, along with custom-built databases. Subsequently, data preprocessing was conducted to obtain the final data matrix used for subsequent analysis.

The data analysis includes the following components: Univariate statistical analysis (volcano plot), multivariate statistical analysis, differential metabolite screening, analysis of the correlation of differential metabolites, and KEGG pathway analysis^17–19^.

### Determination of *Ganoderma lucidum* fruiting body yield and active ingredients

Fruiting body yield determination: The yield of *Ganoderma lucidum* fruiting bodies was statistically recorded, with a sample size of 150 plants and 3 replicates set. Diameter of the fruiting body: The diameter of the fruiting body was measured using a vernier caliper, with 3 replicates set.

Determination of soluble total sugars: Accurately weigh 1.00 g of sample, add 1 mL of sterile water, sonicate for 10 min, and centrifuge at 12,000 rpm for 10 min. Pipette 0.4 mL of the supernatant into a new test tube and dilute it 10 times with ultrapure water. Then, in the test tube, mix the diluted sample solution with phenol and concentrated sulfuric acid, react, measure the absorbance at 490 nm, and calculate the total sugar content of the sample based on the standard curve, with 3 replicates set.

Ganoderma triterpene determination: Accurately weigh 0.10 g of sample, place it in a 100 mL volumetric flask, dissolve it in ethyl acetate, sonicate for 30 min, dilute to the mark, filter, and precisely pipette 1.0 mL of the filtrate into a 10 mL test tube. Evaporate to dryness on a water bath at 100 °C, add 0.20 mL of 5% vanillin-acetic acid and 1.0 mL of perchloric acid, mix, heat in a 60 °C water bath for 20 min, cool in an ice bath for 3 min, then add 5.00 mL of acetic acid, mix, and let stand at room temperature for 15 min. Measure the absorbance of the sample solution at a wavelength of 548 nm using a spectrophotometer, calculate the triterpene content of the sample based on the standard curve, with 3 replicates set.

### The method of soil bulk density and soil moisture content

Firstly, collect a certain amount of dry soil sample, weigh and record its mass (m1). Then place the soil sample in a container and compact it using a standard method, weigh the container again and record its mass (m2). Finally, calculate the soil bulk density according to the formula (m2 − m1) /V, where V is the volume of the container.

Drying Method: Take a certain amount of soil sample and place it in a heating oven or dryer at a high temperature for a period of time until all the moisture in the soil evaporates completely. Then weigh the soil sample again and record its mass (m1). Finally, calculate the moisture content according to the formula (m0 − m1)/m1*100%, where m0 is the initial mass of the soil sample.

## Results and analysis

### Impact of continuous cultivation of Ganoderma on soil physicochemical factors and enzyme activity

**Figure 1:**
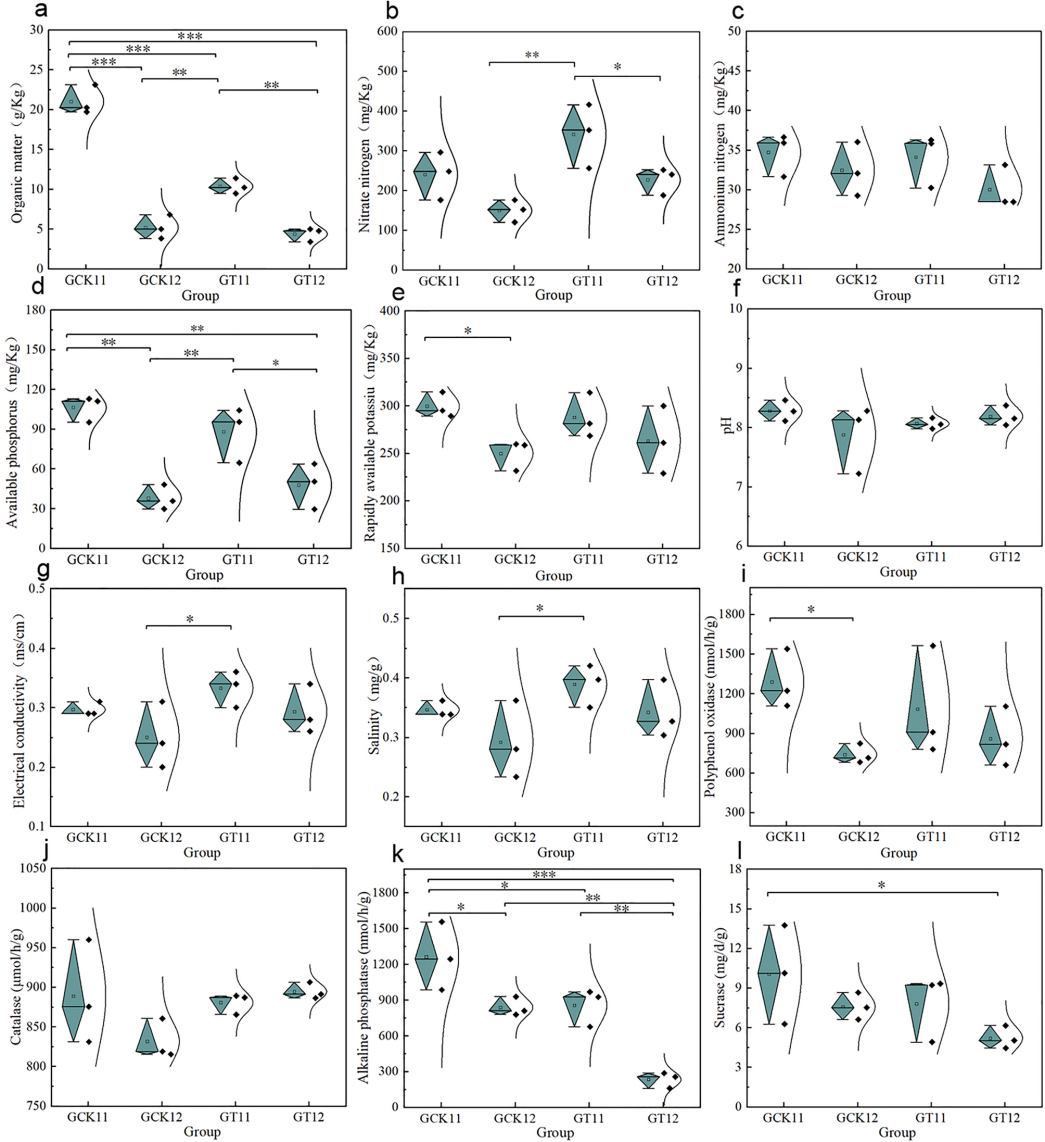
The effects of continuous cultivation of *Ganoderma lucidum* on soil physicochemical factors and enzyme activities. Where *P* < 0.001 is marked as ***, *P* < 0.01 is marked as **, and *P* < 0.05 is marked as *. Organic matter content (**a**), nitrate nitrogen content (**b**), ammonium nitrogen content (**c**), available phosphorus content (**d**), available potassium content (**e**), pH value (**f**), EC value (**g**), salt content (**h**), polyphenol oxidase content (**i**), peroxidase content (**j**), alkaline phosphatase content (**k**), sucrose content (**l**).

Continuous cultivation of *Ganoderma lucidum* has a significant impact on the physicochemical factors of the soil. Regarding the organic matter content (Fig. 1a), GCK11 exhibited a significantly higher level compared to GCK12 and GT11, as well as GT12 (*P* < 0.001). This indicates that the organic matter content in the surface soil (0–15 cm) is significantly higher than that in the deep soil (15–30 cm) (*P* < 0.01). Continuous cultivation of *Ganoderma lucidum* reduces the organic matter content in the soil, especially in the surface soil (0–15 cm). In terms of soil nitrate nitrogen content (Fig. 1b), GT11 demonstrated significantly higher levels compared to GCK12 (*P* < 0.01) and GT12 (*P* < 0.05).This suggests that the nitrate nitrogen content in the surface soil (0–15 cm) is higher than that in the deep soil (15–30 cm), and continuous cultivation of *Ganoderma lucidum* increases the nitrate nitrogen content in the soil. There is no significant differences among them (*P* > 0.05) (Fig. 1c). The content of ammonium nitrogen in the surface soil (0–15 cm) is higher than that in the deep soil (15–30 cm). Continuous cultivation of *Ganoderma lucidum* shows a decreasing trend in ammonium nitrogen content in the soil. In terms of available phosphorus content (Fig. 1d), GCK11 is significantly higher than GCK12 and GT12 (*P* < 0.01), and GT11 significantly higher than GCK12 (*P* < 0.01) and GT12 (*P* < 0.05). This indicates that the available phosphorus content in the surface soil (0–15 cm) is significantly higher than that in the deep soil (15–30 cm). The content of available phosphorus in the surface soil reduced by continuous cultivation (0–15 cm), but increases it in the deep soil (15–30 cm). Regarding available potassium content (Fig. 1e), and GCK11 is significantly higher than GCK12 (*P* < 0.05), indicating that the available potassium content in the surface soil (0–15 cm) is higher than that in the deep soil (15–30 cm), which is more significant before continuous cultivation of *Ganoderma lucidum*. The available potassium content in the soil under continuous cultivation of *Ganoderma lucidum* shows a decreasing trend. Regarding soil pH (Fig. 1f), which indicate that with continuous cultivation of *Ganoderma lucidum*, the pH of the surface soil (0–15 cm) shows a decreasing trend, while the pH of the deep soil (15–30 cm) tends to increase to some extent. Regarding soil EC content (Fig. 1g), GT11 is significantly higher than GCK12 (*P* < 0.05) in soil EC content indicating that with continuous cultivation of *Ganoderma lucidum*, the electrical conductivity in the soil shows an increasing trend. Regarding soil salt content (Fig. 1h), GT11 is significantly higher than GCK12 (*P* < 0.05) in soil salt content, indicate thatcontinuous cultivation of *Ganoderma lucidum*, the content of salt in soil shows an increasing trend, consistent with the trend of soil electrical conductivity changes.

Through the detection of soil enzyme activity, the results showed that GCK11 is significantly higher than GCK12 (*P* < 0.05). Which indicates that the content of polyphenol oxidase in the surface soil (0–15 cm) is higher than that in the deep soil (15–30 cm). Continuous cultivation of *Ganoderma lucidum* leads to a decreasing trend of polyphenol oxidase in the surface soil (0–15 cm) while showing an increasing trend in the deep soil (15–30 cm). there is no significant differences among them (*P* > 0.05) (Fig. 1j). Continuous cultivation of *Ganoderma lucidum* leads to an increasing trend of catalase in the deep soil (15–30 cm) while showing almost no change in the surface soil (0–15 cm). The values of GT11 is significantly higher than GT12 (*P* < 0.01), significantly lower than GCK11 (*P* < 0.05), GT12 significantly lower than GCK11 (*P* < 0.001) and GCK12 (*P* < 0.01), and GCK11 significantly higher than GCK12 (*P* < 0.05). This indicates that the content of alkaline phosphatase in the surface soil (0–15 cm) is significantly higher than that in the deep soil (15–30 cm) (*P* < 0.05). Continuous cultivation of *Ganoderma lucidum* leads to a significant decrease in alkaline phosphatase content in both surface soil and deep soil (*P* < 0.05).Fig. 1l showed that GCK11 significantly higher than GT12. This indicates that the content of sucrase in the surface soil (0–15 cm) is higher than that in the deep soil (15–30 cm). Continuous cultivation of *Ganoderma lucidum* leads to a decreasing trend in sucrase content in both surface soil (0–15 cm) and deep soil (15–30 cm).

Analysis of soil moisture content and soil bulk density under continuous cultivation of *Ganoderma lucidum* revealed that there is no significant differences in both soil moisture content and bulk density (*P* > 0.05). However, the soil moisture content in the surface layer (0–15 cm) was significantly higher than that in the deeper layer (15–30 cm) (*P* < 0.01), while the soil bulk density in the surface layer (0–15 cm) was significantly lower than that in the deeper layer (15–30 cm) (*P* < 0.05) (Figure S1).

### The continuous cultivation of *Ganoderma lucidum* affects the yield and active ingredients of its fruiting bodies

**Figure 2:**
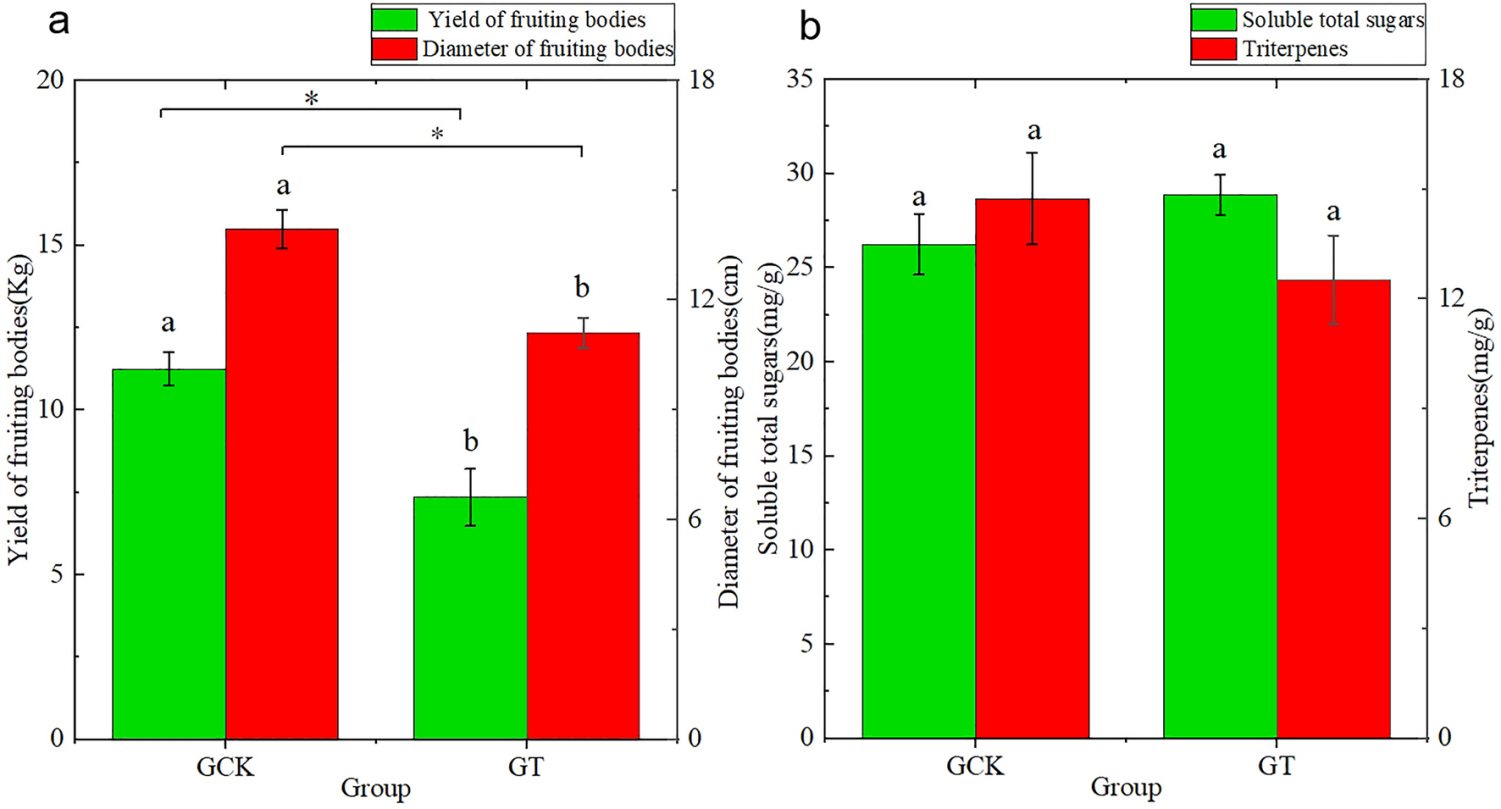
The continuous cultivation of *Ganoderma lucidum* affects the yield and active ingredients of its fruiting bodies.

Continuous cultivation of *Ganoderma lucidum* has had a significant impact on the yield and active ingredients of *Ganoderma lucidum* fruiting bodies. The yield and diameter of *Ganoderma lucidum* fruiting bodies in the GCK group were significantly greater than those in the GT group (*P* < 0.05). The soluble polysaccharide content in the GCK group was lower than that in the GT group, while in terms of triterpene content, the GCK group was higher than the GT group. However, there was no significant difference in both results (*P* > 0.05) (Fig. 2).

### *Ganoderma lucidum* continuous cropping substrate metabolomics differential analysis

#### PCA analysis

**Figure 3:**
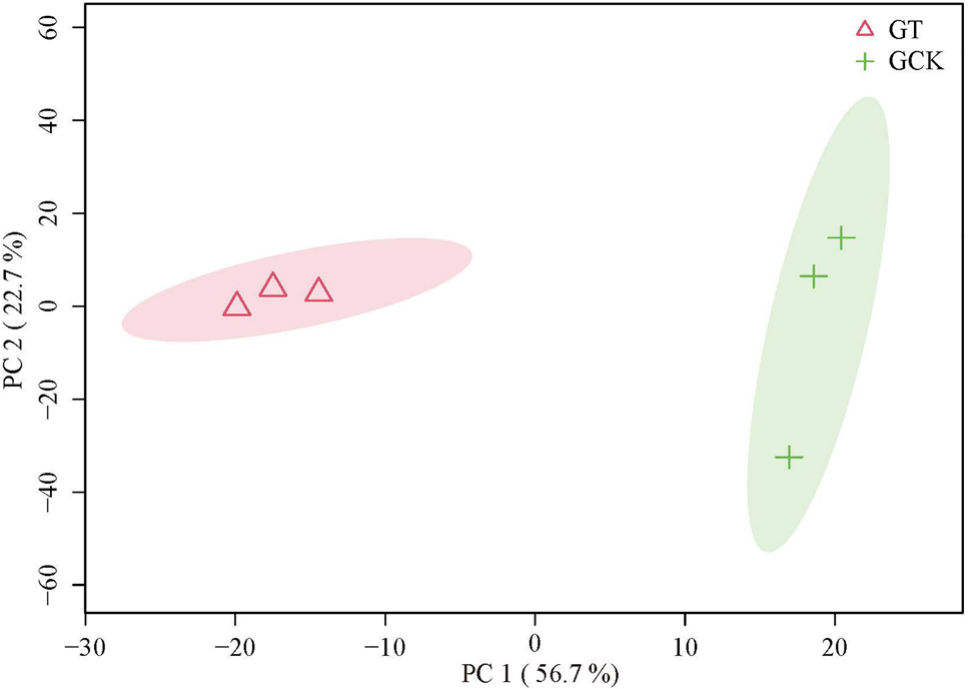
PCA score plot of GT group versus GCK group.

From Fig. 3, it can be observed that PC1 contributes 56.7% and PC2 contributes 22.7% to the variance, resulting in a cumulative contribution rate of 0.79 for the model. Therefore, the PCA model exhibits good fitness. Additionally, all samples are within the 95% confidence interval. Subsequently, the GT and GCK samples are distinctly clustered into two groups, indicating significant compositional differences between the groups. This suggests that further investigation of the inter-group components is warranted.

#### Orthogonal partial least squares discriminant analysis (OPLS-DA) analysis

**Figure 4:**
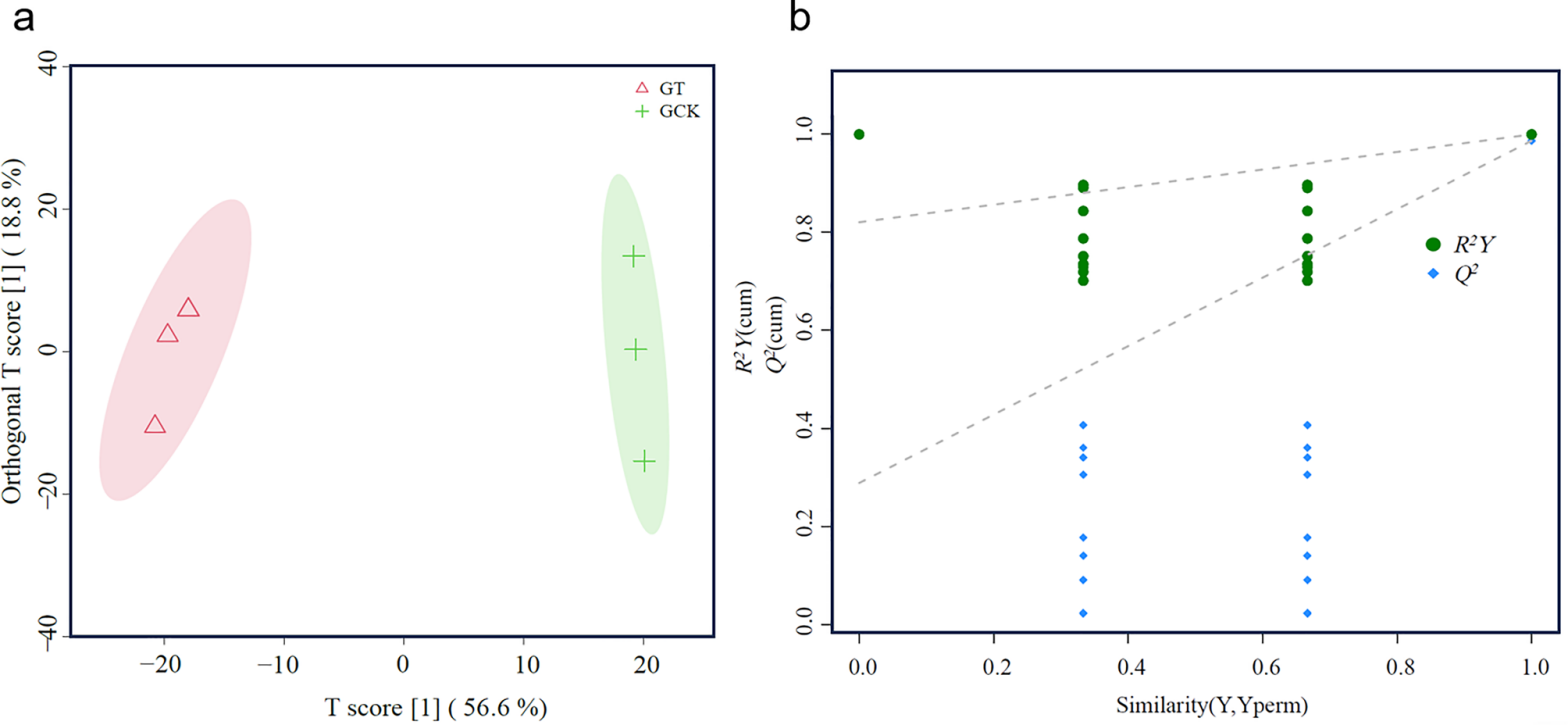
OPLS-DA score plot (**a**) and permutation test plot (**b**) for the comparison between the GT group and the GCK group.

To obtain more precise results, Orthogonal partial least squares discriminant analysis (OPLS-DA) was employed to establish a relationship model between metabolite expression levels and sample categories. This approach amplifies the differences between groups while minimizing differences within groups, filtering out irrelevant information for classification, and enabling rapid and accurate analysis of inter-group differences. As shown in Fig. 4a, all samples fall within the 95% confidence interval, indicating a good predictive capability of the model as most data points are simulated within. Samples from GT and GCK are distributed on the right and left sides of the confidence interval, respectively, indicating a significant difference in components between the two samples, making them suitable for differential metabolite identification. Model evaluation parameters obtained from seven cycles of cross-validation are presented in Fig. 4b, showing a cumulative predictive rate *Q*^*2*^ = 0.29 and *R*^*2*^*Y* = 0.82. All *Q*^*2*^ values from left to right remain lower than the original blue *Q*^*2*^ point, indicating good stability and effectiveness of the model.

### Differential metabolite screening

**Figure 5:**
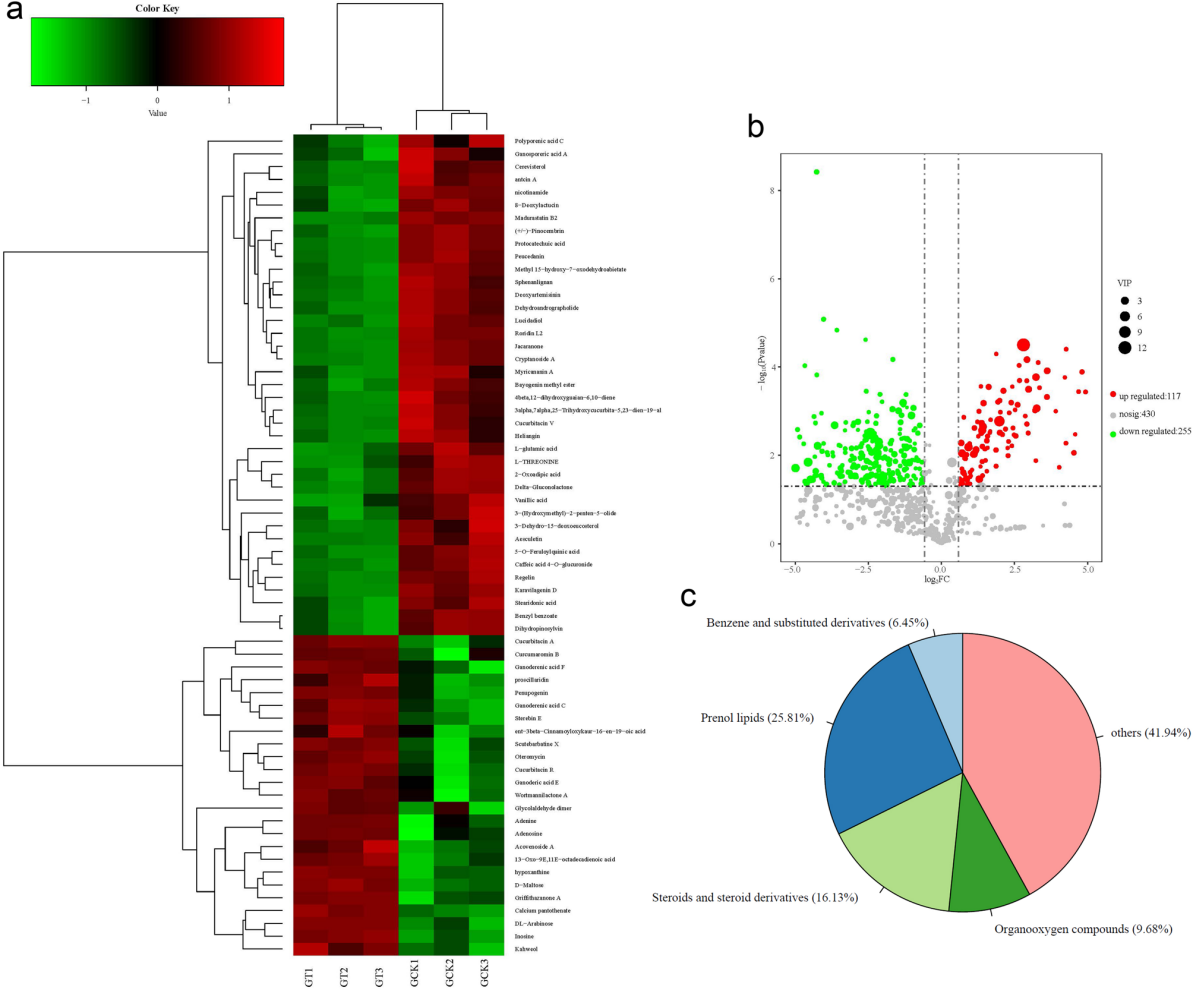
Differential metabolite heatmap (**a**), volcano plot (**b**), and classification pie chart (**c**).

Based on the OPLS-DA results, differential metabolite analysis between the GT and GCK groups of *Ganoderma lucidum* fruiting bodies was conducted. Substances with VIP values greater than 1 and *P*-values less than 0.05 were used to generate the volcano plot of metabolites (Fig. 5b). Among the metabolites, 117 were upregulated and 255 were downregulated in the GT group compared to the GCK group. Considering the previous analyses, the final differential substances were obtained. A clustered heatmap analysis was performed on the differential substances of the two groups, as shown in Fig. 5a. A total of 64 significantly different metabolites were identified in the metabolites of *Ganoderma lucidum* fruiting bodies between the GT and GCK groups, accounting for 7.98% of all identified metabolites. Among these 64 differential metabolites, 39 components showed significantly higher relative abundance in the GCK group compared to the GT group, while 25 components showed significantly lower relative abundance in the GCK group. These metabolites mainly belonged to four categories: Prenol lipids, steroids and steroid derivatives, organooxygen compounds, and benzene and substituted derivatives, accounting for 25.81%, 16.13%, 9.68%, and 6.45% of all identified metabolites, respectively (Fig. 5c).

### Differential metabolite analysis

**Figure 6:**
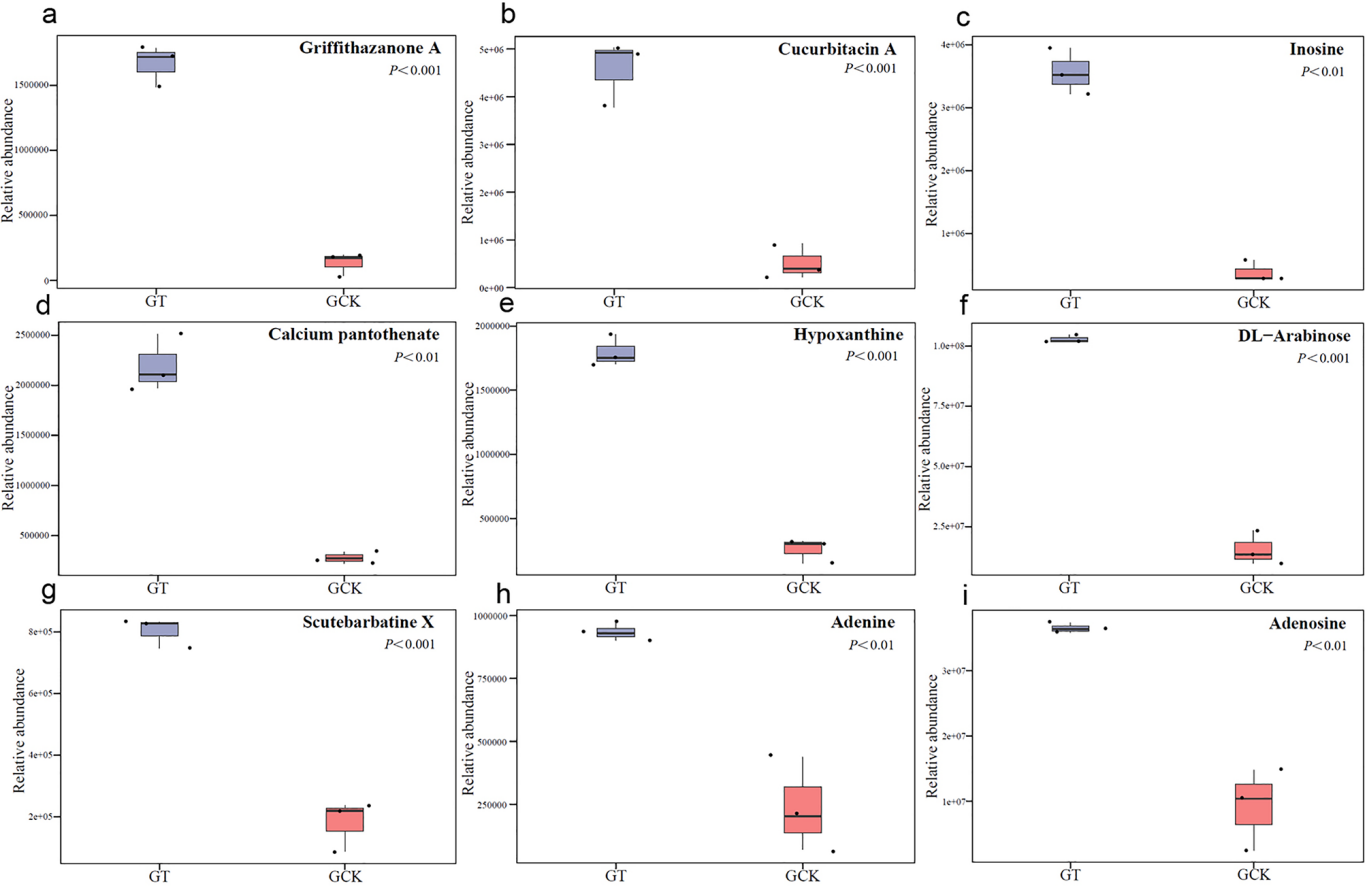
Substances with higher content in GT among differential metabolites. Griffithazanone A content (**a**), cucurbitacin A content (**b**), inosine content (**c**), calcium pantothenate content (**d**), hypoxanthine content (**e**), DL-arabinose content (**f**), scutebarbatine X content (**g**), adenine content (**h**), adenosine content (**i**).

The research results indicate that substances with relatively higher abundance in GT (Fig. 6) include griffithazanone A, cucurbitacin A, inosine, calcium pantothenate, hypoxanthine, DL-arabinose, scutebarbatine X, adenine, and adenosine. These substances exhibit significantly higher differential multiples compared to GCK, with values of 12.35, 9.62, 9.43, 7.93, 7.57, 6.99, 4.35, 4.00, and 3.95, respectively. They mainly belong to categories such as prenol lipids, steroids and steroid derivatives, and organooxygen compounds.

**Figure 7:**
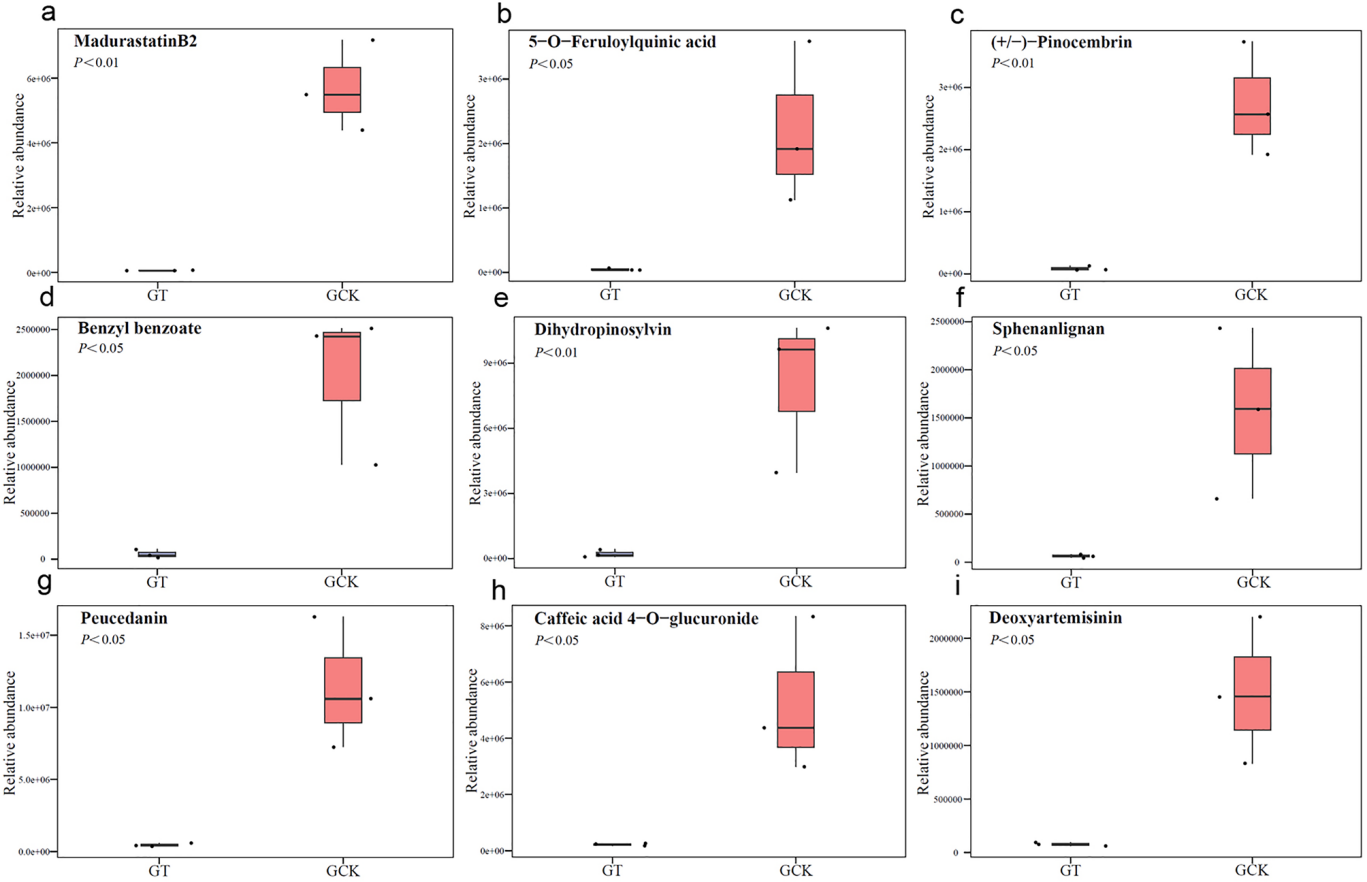
Substances with higher content in GCK among differential metabolites. Madurastatin B2 content (**a**), 5-O-feruloylquinic acid content (**b**), (±)-pinocembrin content (**c**), benzyl benzoate content (**d**), dihydropinosylvin content (**e**), sphenanlignan content (**f**), peucedanin content (**g**), caffeic acid 4-O-glucuronide content (**h**), deoxyartemisinin content (**i**).

Substances with relatively higher abundance in GCK (Fig. 7) include madurastatin B_2_, 5-O-feruloylquinic acid, (±)-pinocembrin, benzyl benzoate, dihydropinosylvin, sphenanlignan, peucedanin, caffeic acid 4-O-glucuronide, and deoxyartemisinin. These substances exhibit significantly higher differential multiples compared to GT. They mainly belong to categories such as prenol lipids, benzene and substituted derivatives, and organooxygen compounds.

### Differential metabolite pathway analysis, KEGG pathway enrichment analysis, and MetPA analysis

**Figure 8:**
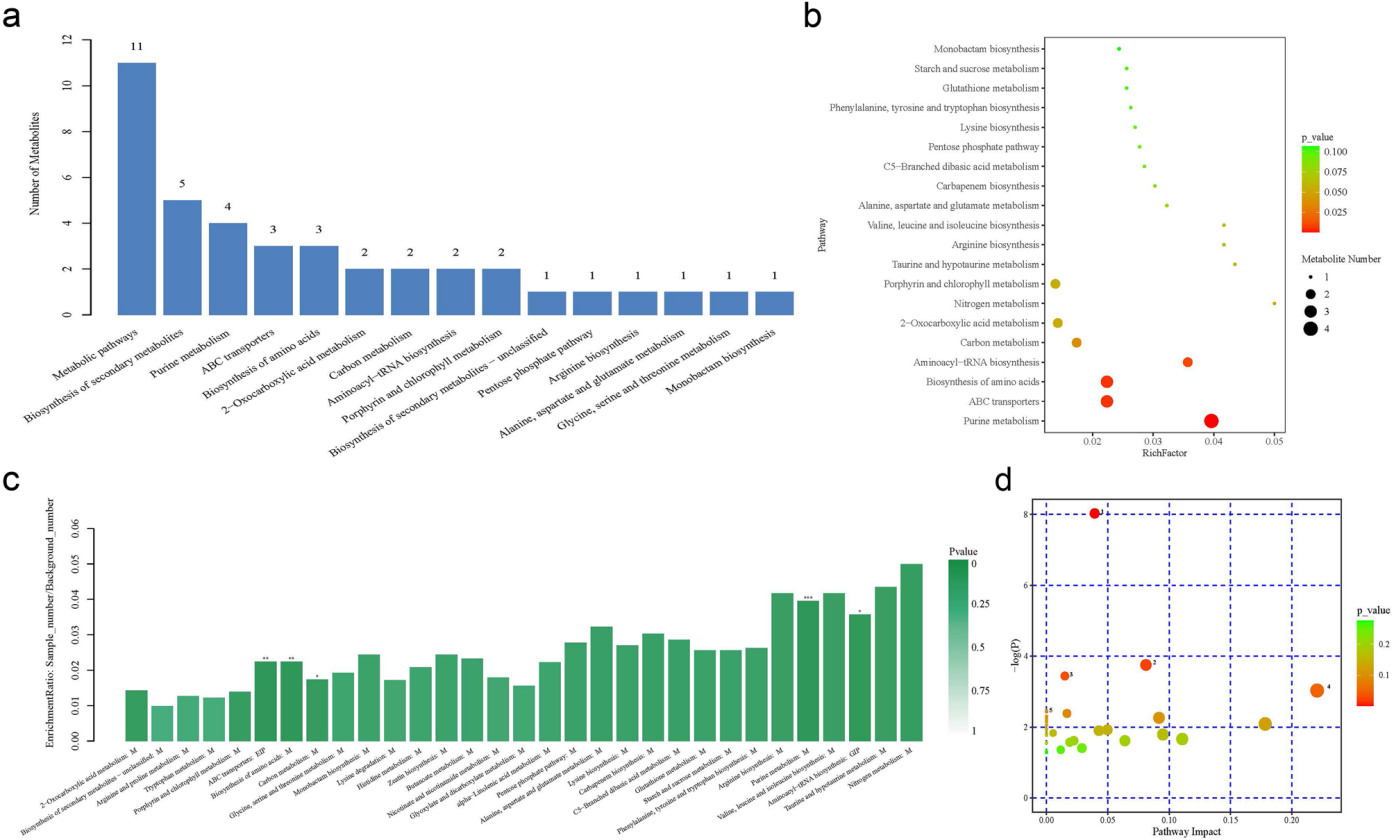
Differential Metabolite Pathway Analysis (**a**), KEGG Pathway Enrichment Analysis (**b**,**c**), and MetPA Analysis (**d**).

Through the KEGG database, pathway enrichment analysis was conducted on differential metabolites, with a total of 33 pathways detected. Among the 64 significantly different metabolites identified, 58 were annotated in KEGG, mainly distributed in 33 metabolic pathways. GCK showed more and complex metabolic pathways compared to GT. As shown in Fig. 8a, the top 5 enriched pathways were: Metabolic pathways, biosynthesis of secondary metabolites, purine metabolism, ABC transporters, and biosynthesis of amino acids, including 11, 5, 4, 3, and 3 differential metabolites, respectively.

As shown in Fig. 8b and c, the pathways with high enrichment rates were nitrogen metabolism, taurine and hypotaurine metabolism, arginine biosynthesis, valine, leucine and isoleucine biosynthesis, and purine metabolism. Significantly enriched pathways included purine metabolism (*P* < 0.001), ABC transporters (*P* < 0.01), biosynthesis of amino acids (*P* < 0.01), aminoacyl-tRNA biosynthesis (*P* < 0.05), and carbapenem biosynthesis (*P* < 0.05), indicating that continuous cultivation of *Ganoderma lucidum* significantly affected the above 10 metabolic pathways in the fruiting bodies.

As shown in Fig. 8d, the significance of metabolite involvement in enriched pathways was as follows: Purine metabolism, aminoacyl-tRNA biosynthesis, aminobenzoate degradation, D-glutamine and D-glutamate metabolism, and porphyrin and chlorophyll metabolism. The relative importance of these 5 metabolites involved in the pathways was D-glutamine and D-glutamate metabolism, aminoacyl-tRNA biosynthesis, purine metabolism, aminobenzoate degradation, and porphyrin and chlorophyll metabolism, respectively.

### Analysis of the correlation between differential metabolites of *Ganoderma lucidum* fruiting bodies and soil physicochemical factors

**Figure 9:**
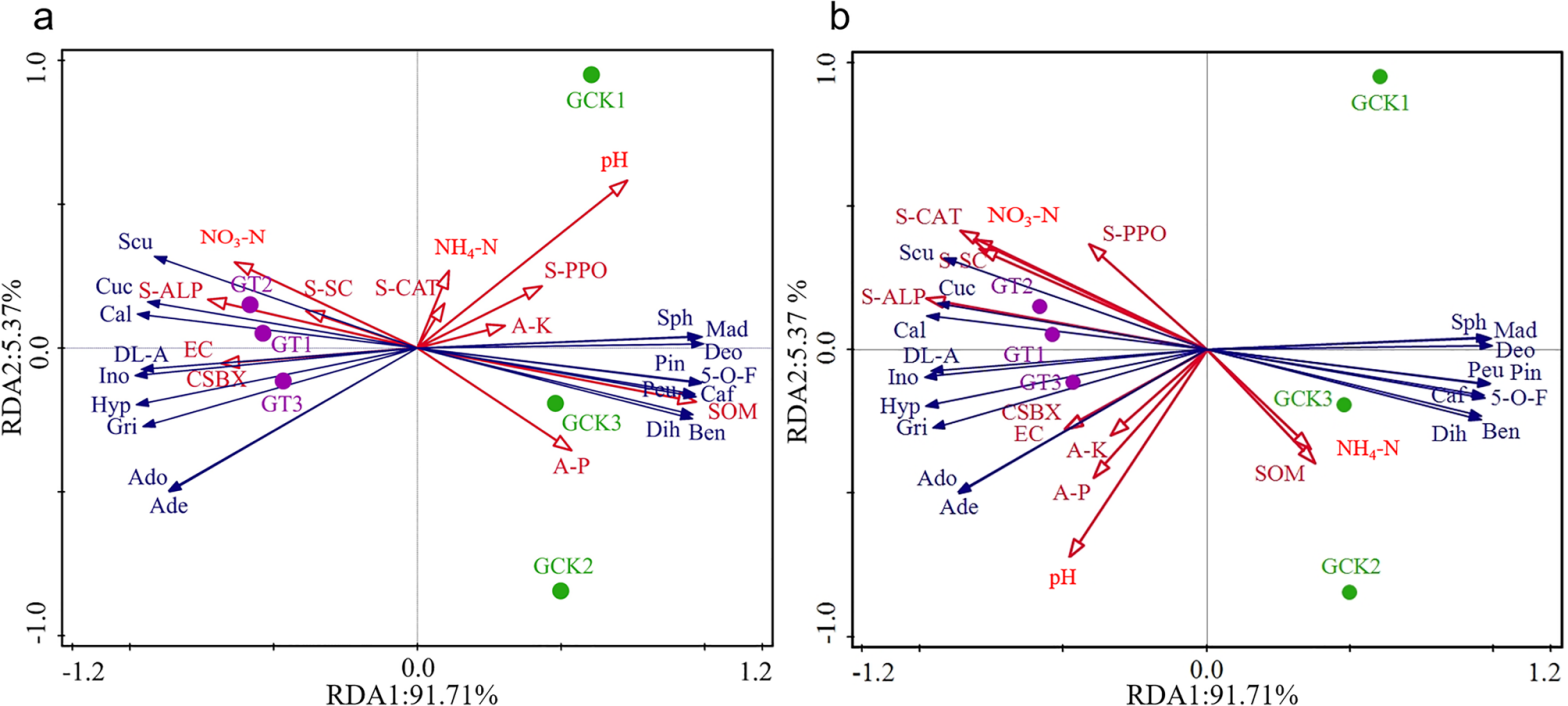
RDA analysis of differential metabolites and soil physicochemical factors influenced by *Ganoderma lucidum*. RDA analysis of differential metabolites and surface soil physicochemical factors influenced by *Ganoderma lucidum* (**a**), RDA analysis of differential metabolites and deep soil physicochemical factors influenced by *Ganoderma lucidum* (**b**). Gri: Griffithazanone A; Cuc: Cucurbitacin A; Ino: Inosine; Cal: Calcium pantothenate; Hyp: Hypoxanthine; DL-A: DL-Arabinose; Scu: Scutebarbatine X; Ade: Adenine; Ado: Adenosine; Mad: Madurastatin B_2_; 5-O-F: 5-O-Feruloylquinic acid; Pin: (±)-Pinocembrin; Ben: Benzyl benzoate; Dih: Dihydropinosylvin; Sph: Sphenanlignan; Peu: Peucedanin; Caf: Caffeic acid 4-O-glucuronide; Deo: Deoxyartemisinin. SOM: Soil Organic Matter; NH_4_-N: Ammonium Nitrogen; NO_3_-N: Nitrate Nitrogen; A-P: Available Phosphorus; A-K: Available Potassium; EC: Electrical Conductivity; pH: Potential of Hydrogen; CSBX: Soil Salinity; S-PPO: Soil Polyphenol Oxidase; S-CAT: Soil Catalase; S-SC: Soil Sucrase; S-ALP: Soil Alkaline Phosphatase.

RDA analysis was conducted by Canoco 5.0 software to explore the relationship between the differential metabolites of *Ganoderma lucidum* fruiting bodies and the physicochemical factors of surface soil (0–15 cm) and subsurface soil (15–30 cm) (Fig. 9). The explanatory percentages of axes one and two were 91.71% and 5.37%, respectively, with a cumulative explanatory percentage of 97.08%, indicating a good reflection of the relationship between the differential metabolites of *Ganoderma lucidum* fruiting bodies and soil physicochemical factors. The length of the arrows represents the intensity of the influence of environmental factors on the microbial community structure, with longer arrows indicating stronger correlations. Acute angles between arrow connections indicate positive correlations, while obtuse angles indicate negative correlations. Figure 9a shows that the differences in *Ganoderma lucidum* fruiting bodies metabolites in surface soil (0–15 cm) are significantly influenced by soil pH, SOM (soil organic matter), available phosphorus (A-P), soil alkaline phosphatase (S-ALP), and nitrate nitrogen (NO_3_-N). Specifically, pH, SOM, and A-P show positive correlations with sphenanlignan, madurastatin B_2_, deoxyartemisinin, (±)-pinocembrin, 5-o-feruloylquinic acid, caffeic acid 4-O-glucuronide, peucedanin, dihydropinosylvin, and benzyl benzoate, while they exhibit negative correlations with scutebarbatine X, cucurbitacin A, calcium pantothenate, DL-arabinose, inosine, hypoxanthine, griffithazanone A, adenosine, and adenine. However, S-ALP and NO_3_-N show opposite correlations. Figure 9b indicates that in subsurface soil (15–30 cm), the differential metabolites of *Ganoderma lucidum* fruiting bodies are significantly influenced by soil alkaline phosphatase (S-ALP), soil catalase (S-CAT), pH, nitrate nitrogen (NO_3_-N), and soil catalase (S-SC). Specifically, S-ALP, S-CAT, pH, NO_3_-N, and S-SC show positive correlations with scutebarbatine X, cucurbitacin A, calcium pantothenate, DL-arabinose, inosine, hypoxanthine, griffithazanone A, adenosine, and adenine, while they exhibit negative correlations with sphenanlignan, madurastatin B_2_, deoxyartemisinin, (±)-pinocembrin, 5-O-feruloylquinic acid, caffeic acid 4-O-glucuronide, peucedanin, dihydropinosylvin, and benzyl benzoate.

## Discussion

Long-term continuous cropping leads to soil nutrient imbalance, which directly or indirectly affects the microbial community structure and metabolic products in the soil^7,20,21^. This study investigated the physicochemical factors of surface soil (0–15 cm) and subsoil (15–30 cm) in continuous cultivation of *Ganoderma lucidum* (Fig. 1). The results indicated that the organic matter content in the surface soil (0–15 cm) was significantly higher than that in the subsoil (15–30 cm) (*P* < 0.01). Continuous cultivation of *Ganoderma lucidum* reduced the organic matter content in the soil, particularly in the surface soil (0–15 cm), consistent with previous studies suggesting that continuous cropping accelerates organic matter loss from the soil^22^. Nitrate nitrogen and ammonium nitrogen content in the surface soil (0–15 cm) were higher than those in the subsoil (15–30 cm). The nitrate nitrogen content in the soil showed an increasing trend, while the ammonium nitrogen content showed a decreasing trend in continuous cultivation of *Ganoderma lucidum*. The nitrification process in the soil, converting ammonium nitrogen into nitrate nitrogen, might be inhibited in continuous cultivation of *Ganoderma lucidum*^23^. The available phosphorus content in the surface soil (0–15 cm) was significantly higher than that in the subsoil (15–30 cm), which could be attributed to the loose structure of soil aggregates in the surface soil favoring phosphorus adsorption. Continuous cultivation of *Ganoderma lucidum* reduced the available phosphorus content in the surface soil (0–15 cm), consistent with previous findings that the available phosphorus content decreases with increasing years of continuous cropping^24^. However, this study found an increase in the available phosphorus content in the subsoil (15–30 cm) under continuous cultivation of *Ganoderma lucidum*, possibly due to the relatively drier conditions of the surface soil in the later stages of Ganoderma cultivation. The available potassium content in the surface soil (0–15 cm) was higher than that in the subsoil (15–30 cm), and it showed a decreasing trend in continuous cultivation of *Ganoderma lucidum*. Continuous cultivation of *Ganoderma lucidum* led to a decreasing trend in pH in the surface soil (0–15 cm), while there was a slight increasing trend in pH in the subsoil (15–30 cm). Electrical conductivity (EC) and salt content in the soil showed an increasing trend under continuous cultivation of *Ganoderma lucidum*, consistent with previous findings that salt content generally increases in soils under continuous cropping^25^.

Soil enzyme activity serves as a crucial indicator of soil fertility, quality, and health. Soil enzymes interact with the soil environment, indirectly influencing soil nutrient levels^26^. Results from soil enzyme activity assays indicate that the content of soil polyphenol oxidase (S-PPO) in the surface soil (0–15 cm) is higher than that in the subsoil (15–30 cm) (Fig. 2). Continuous cultivation of *Ganoderma lucidum* leads to a decreasing trend of S-PPO in the surface soil (0–15 cm) and an increasing trend in the subsoil (15–30 cm). Soil polyphenol oxidase can oxidize aromatic compounds in the soil into quinones, which react with substances such as proteins, sugars, and amino acids in the soil, completing the cycle of aromatic compounds in the soil and simultaneously repairing the soil environment^27^. This suggests that the self-repair capability of the surface soil deteriorates with continuous cultivation of *Ganoderma lucidum*. In continuous cultivation of *Ganoderma lucidum*, the trend of soil catalase (S-CAT) in the subsoil (15–30 cm) shows an increasing trend, while there is almost no change in the surface soil (0–15 cm). This may be attributed to the pH decrease in the surface soil inhibiting enzyme activity, while the pH increase in the subsoil promotes enzyme activity^28^. Soil catalase can reduce the harm of excessive accumulation of hydrogen peroxide in the soil^29^, indicating a weakening of the surface soil’s ability to reduce the harm of excessive accumulation of hydrogen peroxide. The content of soil alkaline phosphatase (S-ALP) and soil sucrase (S-SC) in the surface soil (0–15 cm) is higher than that in the subsoil (15–30 cm). Continuous cultivation of *Ganoderma lucidum* leads to a decreasing trend of S-ALP and S-SC in both surface and subsoil, with a significant decrease in S-ALP (*P* < 0.05). This indicates that the distribution of alkaline phosphatase and sucrase activity in the soil profile decreases from the surface to the subsoil. Alkaline phosphatase can accelerate the conversion rate of organic phosphorus^30^, and the decrease in alkaline phosphatase content affects the decomposition efficiency of organic phosphorus in the soil, thereby affecting the content of available phosphorus in the soil, especially in the surface soil. Soil sucrase activity can serve as an important indicator of soil fertility^31^. The decrease in sucrase indicates a decrease in soil fertility with continuous cultivation of *Ganoderma lucidum*, consistent with the trend of organic matter changes mentioned above.

Continuous cropping not only affects the metabolic products of soil microorganisms^32^, but also influences the metabolic products of subsequently grown products. A total of 64 significantly different metabolites were identified in the metabolic products of *Ganoderma lucidum* fruiting bodies from both the GT and GCK groups (Fig. 5). Among these 64 differential metabolites, 39 were found to have significantly higher relative contents in the GCK group compared to the GT group, while 25 showed significantly lower relative contents. Although the strains are of the same Ganoderma species, the effects of continuous cropping have led to differences in both the types and quantities of metabolites in the two batches of *Ganoderma lucidum* fruiting bodies. Notably, among the differential metabolites, there were more types with higher relative contents in the fruiting bodies harvested in the first year of cultivation (GCK) compared to those harvested in the second year (GT), and the differences in relative contents were also larger. Among the substances with higher relative contents in GCK, madurastatin B_2_, 5-O-feruloylquinic acid, (±)-pinocembrin, benzyl benzoate, dihydropinosylvin, sphenanlignan, peucedanin, caffeic acid 4-O-glucuronide, and deoxyartemisinin were notable. These substances exhibited higher differential multiples compared to GT, with multiples of 68.34, 46.64, 33.73, 32.22, 31.76, 24.41, 23.56, 22.30, and 19.68, respectively. They mainly belong to four categories: Prenol lipids, steroids and steroid derivatives, organooxygen compounds, and benzene and substituted derivatives. Among these substances, 5-O-feruloylquinic acid, classified as organooxygen compounds, possesses various biological activities such as antibacterial, antioxidant, antimutagenic, antitumor, and antiviral activities^33^. (±)-Pinocembrin, a type of flavonoid, exhibits pharmacological effects including the treatment of cardiovascular and cerebrovascular diseases, anti-inflammatory, antibacterial, antioxidant, and inhibition of testosterone 5a-reductase activity^34^. Dihydropinosylvinin has strong antibacterial activity against *Bacillus cereus*, *Staphylococcus aureus*, *Pseudomonas aeruginosa*, and *Escherichia coli*, as well as certain antioxidant activity^35^. Peucedanin shows promising advantages in the design and development of selective antibacterial triazole derivatives^36^. Caffeic acid 4-O-glucuronide exhibits anti-rheumatic effects^37^, while deoxyartemisinin displays anti-inflammatory and anti-ulcer activities^38^.

The KEGG metabolic pathway analysis provides us with insights into understanding the differences in Ganoderma metabolomes. According to the results, GCK has more and more complex metabolic pathways compared to GT, indicating a potentially more significant role of GCK in Ganoderma metabolism. Moreover, the top 5 enriched pathways are Metabolic pathways, Biosynthesis of secondary metabolites, Purine metabolism, ABC transporters, and Biosynthesis of amino acids, which are related to the growth, metabolism, and biological activities of Ganoderma. Further analysis of metabolite involvement in pathways enrichment reveals that Purine metabolism, Aminoacyl-tRNA biosynthesis, Aminobenzoate degradation, D-Glutamine and D-glutamate metabolism, and Porphyrin and chlorophyll metabolism play important roles in Ganoderma metabolism. These pathways involve key biological processes such as nucleic acid synthesis, protein synthesis, and amino acid degradation, further illustrating the complexity and diversity of Ganoderma metabolism. Additionally, the relative importance of differential metabolites in these 5 pathways are D-Glutamine and D-glutamate metabolism, Aminoacyl-tRNA biosynthesis, Purine metabolism, Aminobenzoate degradation, and Porphyrin and chlorophyll metabolism (Fig. 8). These results indicate the prioritization of these metabolic pathways in Ganoderma metabolism, providing valuable clues for further research into the functions of these pathways in Ganoderma growth and metabolism. In summary, KEGG metabolic pathway analysis provides important clues for revealing the characteristics and potential biological functions of Ganoderma metabolomes. Further research will contribute to elucidating the regulatory mechanisms of Ganoderma metabolism and its potential in agricultural production and pharmaceutical applications.

The production of Ganoderma metabolites is largely regulated by soil physicochemical factors and soil enzyme activity, as confirmed by research^39^. In agricultural production, soil quality significantly impacts product quality, emphasizing the importance of in-depth investigation into the relationship between soil environmental factors and Ganoderma metabolites. In surface soil (0–15 cm), we observed significant influences of soil pH, soil organic matter (SOM), total phosphorus content (A-P), and available phosphorus enzyme activity (S-ALP) on Ganoderma metabolites. Specifically, we found that pH, SOM, and A-P were positively correlated with certain metabolites, while negatively correlated with others. This suggests that soil chemical properties play a crucial role in regulating Ganoderma metabolite production. However, S-ALP exhibited an opposite trend to other soil factors, possibly reflecting its unique regulatory mechanism in Ganoderma growth. In deep soil (15–30 cm), we found that soil available phosphorus enzyme activity (S-ALP), catalase activity (S-CAT), soil pH, nitrate nitrogen content (NO_3_-N), and sulfate content (S-SC) significantly influenced Ganoderma metabolic differences. These results further highlight the complex regulatory mechanisms of soil environment on Ganoderma growth and metabolism, particularly at different soil depths (Fig. 9). In summary, the findings of this study deepen our understanding of the effects of soil environment on Ganoderma metabolite production and provide important references for optimizing Ganoderma growth conditions. Future research could further explore the dynamic regulatory mechanisms of different soil factors on Ganoderma metabolite production, and how to utilize this knowledge to enhance Ganoderma production and application.

### Supplementary Information

Supplementary Information.

## Data Availability

All data generated or analysed during this study are included in this published article.
