# Supplementary material for: The impact of continuous cultivation of Ganoderma lucidum on soil nutrients, enzyme activity, and fruiting body metabolites
Source: Sci Rep. 2024 May 2;14:10097. doi: 10.1038/s41598-024-60750-y (PMC11066026; doi:10.1038/s41598-024-60750-y)
Supplement: Supplementary file 1 — Supplementary Information. [file 41598_2024_60750_MOESM1_ESM.pdf]

Supplementary

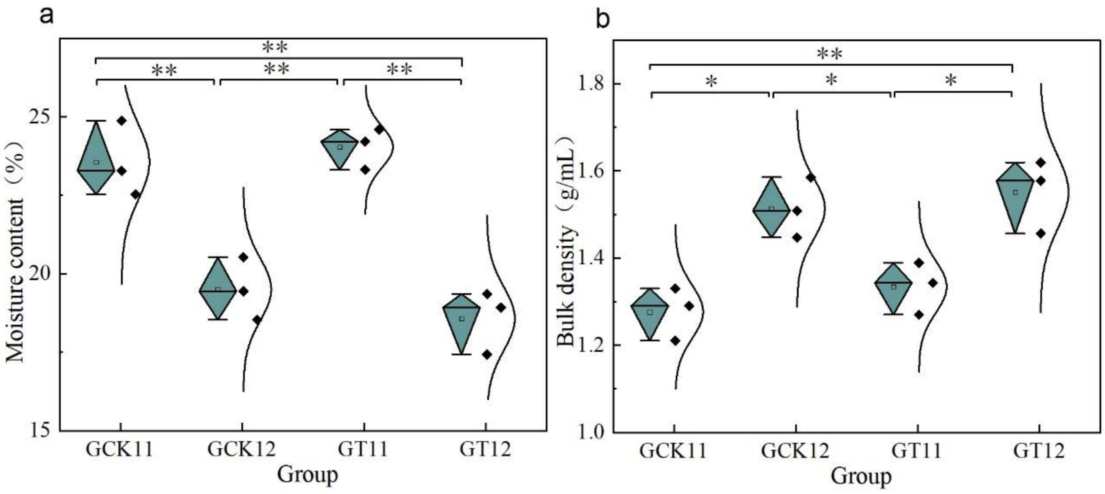

Figure S1 The influence of continuous cultivation of *Ganoderma lucidum* on soil moisture content and bulk density
